# Prevalence and risk factors for myopia and other refractive errors in an adult population in southern India

**DOI:** 10.1111/opo.12447

**Published:** 2018-03-25

**Authors:** Sanil Joseph, Tiruvengada Krishnan, Ravilla D. Ravindran, Giovanni Maraini, Monica Camparini, Usha Chakravarthy, Thulasiraj D. Ravilla, Andrew Hutchings, Astrid E. Fletcher

**Affiliations:** ^1^ Lions Aravind Institute of Community Ophthalmology Aravind Eye Care System Madurai India; ^2^ Aravind Eye Hospital Pondicherry India; ^3^ Aravind Eye Care System Madurai India; ^4^ Sezione di Oftalmologia Dipartimento di Scienze Otorino‐Odonto‐Oftalmologiche e Cervico Facciali Università degli Studi di Parma Parma Italy; ^5^ Dipartimento di Scienze Biomediche, Biotecnologiche e Traslazionali‐S.Bi.Bi.T Università degli Studi di Parma Parma Italy; ^6^ Centre for Vision & Vascular Science School of Medicine, Dentistry and Biomedical Sciences Queen's University Belfast Belfast UK; ^7^ Department of Health Services, Research and Policy London School of Hygiene & Tropical Medicine London UK; ^8^ Faculty of Epidemiology & Population Health London School of Hygiene & Tropical Medicine London UK

**Keywords:** astigmatism, epidemiology, hyperopia, India, myopia, refractive errors

## Abstract

**Purpose:**

To investigate prevalence and risk factors for myopia, hyperopia and astigmatism in southern India.

**Methods:**

Randomly sampled villages were enumerated to identify people aged ≥40 years. Participants were interviewed for socioeconomic and lifestyle factors and attended a hospital‐based ophthalmic examination including visual acuity measurement and objective and subjective measurement of refractive status. Myopia was defined as spherical equivalent (SE) worse than −0.75 dioptres (D), hyperopia as SE ≥+1D and astigmatism as cylinder <−0.5.

**Results:**

The age‐standardised prevalences of myopia, hyperopia and astigmatism were 35.6% (95% CI: 34.7–36.6), 17.0% (95% CI: 16.3–17.8) and 32.6 (29.3–36.1), respectively. Of those with myopia (*n* = 1490), 70% had advanced cataract. Of these, 79% had presenting visual acuity (VA) less than 6/18 and after best correction, 44% of these improved to 6/12 or better and 27% remained with VA less than 6/18. In multivariable analyses (excluding patients with advanced cataract), increasing nuclear opacity score, current tobacco use, and increasing height were associated with higher odds of myopia. Higher levels of education were associated with increased odds of myopia in younger people and decreased odds in older people. Increasing time outdoors was associated with myopia only in older people. Increasing age and female gender were associated with hyperopia, and nuclear opacity score, increasing time outdoors, rural residence and current tobacco use with lower odds of hyperopia. After controlling for myopia, factors associated with higher odds of astigmatism were age, rural residence, and increasing nuclear opacity score and increasing education with lower odds.

**Conclusions:**

In contrast to high‐income settings and in agreement with studies from low‐income settings, we found a rise in myopia with increasing age reflecting the high prevalence of advanced cataract.

## Introduction

Myopia is the most common cause of refractive errors in both children and adults in many countries. Comparisons of adult myopia prevalence across countries are complicated by variations in the age ranges of populations studied, definitions of myopia, prevalence of cataract and, within populations, ancestral heterogeneity, migration and acculturation and secular trends in environmental risk factors.^1^, ^2^, ^3^, ^4^ For example, myopia prevalence in the United States differs by European, African and Hispanic ancestry,^5^ and between Chinese, Malay and Indian ancestry in Singapore.^6^ The pattern of age‐specific rates of myopia also differs between studies. Myopia prevalence has been observed to increase with age in studies in low‐income settings^7^, ^8^, ^9^ and to decrease with age in high‐income settings,^5^, ^10^ while varied patterns, such U shape or inverted J shape distributions, have been reported in other settings, income and population subgroups.^11^, ^12^, ^13^, ^14^, ^15^ Progress has been made in identifying genetic variants for myopia in populations of European or Asian ancestry (primarily Chinese).^16^, ^17^ Of the environmental risk factors, higher education, less time spent outdoors and more time spent in near work activities have been identified as risk factors for myopia primarily in studies from Western^18^, ^19^ and East and Southeast Asian populations^12^, ^13^, ^15^, ^20^ and have been suggested as a reason for the recent increase in the prevalence of myopia in young adults and children.^3^, ^20^

There are limited data for India on myopia prevalence and risk factors in the adult population.^9^, ^21^, ^22^ One study investigated education and reported increasing levels were inversely associated with myopia.^9^ No studies in India have investigated time spent outdoors. We investigated prevalence and risk factors for myopia, hyperopia and astigmatism in the Indian Study of Age‐related Eye Disease (INDEYE), a population‐based study in people aged 40 years and over, with information on education level and time spent outdoors.

## Methods

INDEYE is a population‐based study of people aged 40 years and over in two locations in north and south India. Measurement of refractive errors was collected in all participants in the south India location and therefore forms the basis for the present analysis.

The study sampling has been described in detail elsewhere.^23^ People aged ≥40 years were identified from household enumeration of randomly sampled clusters in south India in the catchment area of Aravind Eye Hospital, Pondicherry (AEH). All persons aged 60 years and over were invited to participate in the study, and a random sample of one in four, stratified by cluster, of those aged 40–59 years was selected. Participants gave full, informed written consent. Illiterate subjects had the information leaflet read to them and provided a thumb impression. The study complied with the guidelines in the Declaration of Helsinki and ethics approval was received from the Indian Council of Medical Research (ICMR), Research Ethics Committees of Aravind Eye Hospital, Pondicherry; London School of Hygiene and Tropical Medicine, London; and Queen's University, Belfast.

### Study procedures

Fieldworkers interviewed participants at home with a structured questionnaire including demographic and socio‐economic data: (quality of house, land ownership, crowding, education, occupation, caste, religion); current and past tobacco use (smoking beedis or cigarettes, chewing tobacco, or inhaling snuff); current and past alcohol use (frequency and type of drink); and type of cooking fuels and stoves.

We asked about current and past work activities including paid and unpaid work and domestic work. For each type of work activity we asked about time spent outdoors (1) in daylight hours, and (2) in the middle of the day, use of protective headgear or glasses and length of time in years that that work period lasted. In addition, we asked the same questions relating to activities in young adulthood (age of 20 years). The purpose of this set of questions was to capture the work activities during early adult life. Within a week of the home interview, participants were brought to AEH for the clinical examination comprising anthropometric measurements including height, an eye examination and blood sample collection. A non‐fasting sample of capillary blood was assessed for glucose (CBG) using a reagent strip test and reflectance meter. In the case of refusal to the hospital‐based examination, participants were re‐contacted at least once and up to three times for people who were unavailable.

### Eye examination

Visual acuity (VA) was tested in each eye separately with the subject wearing habitual spectacles (if any) using the Early Treatment of Diabetic Retinopathy Study (ETDRS) tumbling E chart at a distance of 4 m and recorded as Snellen equivalent (≥4 of 5 letters correctly identified in each row). The ETDRS charts were made of non‐reflective white polystyrene material and were installed in a retro illuminated box (2 × 2 feet). Three fluorescent tube lights of 20 W each placed behind the chart illuminated the chart (luminescence of 150 cd m^−^² or greater). The vision testing started with the top of the chart and continued until a line was reached where more than half the letters (for example, two of four, three of five) were read incorrectly or the patient read all letters on the chart. Uncorrected, presenting VA with participants wearing glasses (if any) and best corrected VA after refractive correction were recorded in each eye. Refractive status was assessed both objectively and subjectively by trained optometrists for all subjects irrespective of the presenting VA. Objective refraction was done using a streak retinoscope to determine the spherical and astigmatism components. This reading was then refined through subjective refraction by testing the vision for distance using the ETDRS chart and by placing the full spherical correction in the trial frame. Jackson's cross cylinder was used to refine the cylindrical axis and cylindrical power. All clinical measurements took place in special study dedicated clinic rooms at AEH. Two optometrists performed VA measurement and refraction and two ophthalmologists performed clinical eye examinations for all study patients which included anterior and posterior segment assessments, using slit lamp biomicroscopy.

Following pupillary dilation using 1% tropicamide, digital slit beam images of the lens were taken using the Topcon SL‐D7 digital photo slit lamp (http://www.topconmedical.com) for nuclear opacities and retroillumination images of the lens using the Neitz CT‐S cataract screener for cortical and posterior subcapsular opacities (http://www.neitz-ophthalmic.com). Digital lens images of each eye were graded according to the Lens Opacities Classification System III (LOCS III)^24^ in 0.1 unit steps up to a maximum of 6.9 for nuclear opacities, and up to a maximum of 5.9 for cortical and posterior subcapsular opacities (PSC). Psuedophakia or aphakia was assessed from the digital images. Nuclear colour was not graded. The training and quality assurance of the photographers and graders has been described in detail elsewhere.^23^

#### Definition and calculation of refractive error

Refractive errors (RE) were quantified in terms of the spherical equivalent. The spherical equivalent of refractive error (SE) was obtained by adding half of the cylindrical value to the spherical value of the refractive error in each eye. The SE for each individual was calculated by taking the mean of SE of both eyes. Participants with aphakia or pseudophakia in either eye were excluded from the analyses. Myopia was defined as SE worse than −0.75 dioptres (D), and subcategorised as low myopia (≤−0.75 to >−3 D), moderate myopia (≤−3 to >−6 D) and high myopia (≤6 D). Hyperopia was defined as SE ≥+1 D.^10^ Astigmatism was defined as a cylinder less than −0.50 (D) in any axis in either eye. We also present the prevalence data using other cut points (SE worse than −0.50 for myopia and SE worse than +0.5 for hyperopia) for comparison with other studies^9^, ^21^ and in supplementary analysis the myopia prevalence excluding those with advanced cataract.

### Data preparation and statistical analysis

We used principal component analysis to derive a socio‐economic status index (SES; based on caste, landholding, type of roof and number of rooms in house) and entered into models as quartiles of the index. Based on the work activities responses, we calculated the average time (daily hours) spent outdoors currently and in the past. In further analyses we also investigated time spent outdoors daily in early adulthood (age 20 years). Diabetes was defined as a random blood sugar of ≥200 mg dL^−1^.^25^ Tobacco use was categorised as never, past and current. We defined type of advanced cataract based on the LOCS III grade in either eye of: ≥4 for nuclear cataract, ≥3 for cortical cataract, ≥2 for posterior subcapsular cataract (PSC). Any type of advanced unoperated cataract was defined as the presence of any of the above definitions or (in a few participants) widespread dense opacities that could not be graded for type.^23^ We used data collected during the household enumeration to compare the participants and non‐participants by age, gender, SES index or education. Presenting and best corrected VA were categorised using the result in the better eye.

The prevalence and 95% confidence intervals of myopia, hyperopia and astigmatism were estimated by 5‐year age groups up to the age of 70 years and over. We age‐standardised the overall and sex‐specific myopia rates using the Tamil Nadu population distribution for the age groups 40+ years.^26^ We used logistic regression to investigate variables previously reported as risk factors for myopia, hyperopia and astigmatism. These included age, gender, rural or urban residence, socio‐economic status, education, height, diabetes, time spent outdoors, and tobacco use. Height was used as a proxy for axial length.^27^ The reference group in these analyses were those with no myopia, no hyperopia and no astigmatism. Since many studies have reported that significant nuclear cataract^2^, ^28^ or PSC^28^ may be the primary reason for myopia, especially in older individuals, or cortical cataract for astigmatism,^14^, ^29^, ^30^ we excluded any participants with any advanced unoperated type of cataract from risk factor analyses for myopia and astigmatism. Even after exclusion of advanced cataract, we retained the LOCS III nuclear opacity score in all risk factor models as an additional control for milder opacities (LOCS III nuclear opacity score <4). We took a similar approach in risk factor analyses of astigmatism by including additionally the LOCS III cortical opacity score (<3). We hypothesised *a priori* that age might modify the association of education or time spent outdoors with myopia or hyperopia reflecting secular trends in higher education in the younger age groups and less influence of nuclear induced myopic shift. We therefore included an interaction term of age (<60 years, vs 60+ years), with education or with time spent outdoors in the logistic regression models.

All analyses took into account the sampling design in the estimation of robust standard errors and corresponding *p*‐values and 95% confidence intervals and the different sample fractions for the younger and older population. Statistical analyses were carried out using Stata 13 software (http://www.stata.com).

## Results

Of the 6053 people sampled for the study, 4351 (71.9%) attended AEH for the clinical examination. Participants were older than non‐participants, 62 years (S.D. = 11) compared to 59 years (S.D. = 13) respectively (*p* < 0.0001) but there was no difference by gender (*p* = 0.8), SES index (*p* = 0.7) or education (*p* = 0.2). Of the 4351 participants, refractive error data were available for 4342. Among these, 1075 had aphakia or pseudophakia in either eye and were excluded from the analyses of prevalence and 1502 had advanced unoperated cataract (any of the following: ≥4 for nuclear cataract, ≥3 for cortical cataract, ≥2 for posterior subcapsular cataract (PSC)) and were excluded from the risk factor analyses.

### Prevalence of myopia, hyperopia and astigmatism

Of 3267 participants without aphakia or pseudophakia, myopia (≤−0.75 D) occurred in 1490, hyperopia (≥+0.5 D) in 880 and astigmatism (cylinder <−0.50 D) in 1403.

**Figure 1:**
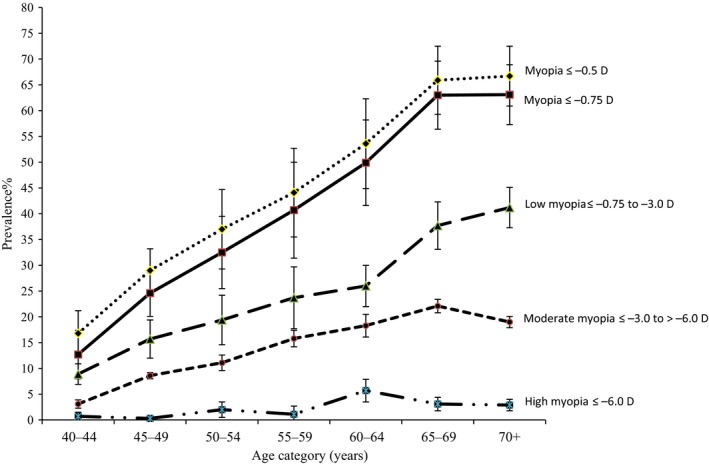
Prevalence of myopia with 95% CI by age for different cut points of spherical equivalents.

The age‐standardised prevalence of myopia ≤−0.75 D was 35.6% (95% CI, 34.7–36.6). The age‐standardised prevalence was slightly higher in women (37.4%, 95% CI 36.1–38.6) than men (33.4%, 95% CI 32.1–34.6). Myopia prevalence increased up to the 60–69 years age group with no additional increase in the 70+ years age group (*Figure* 1). Low myopia showed a pattern of increasing prevalence with increasing age whereas moderate myopia decreased in the oldest age group. There was no clear relationship between age and high myopia but the numbers were small; only 2% had high myopia. After exclusion of those with advanced cataract the prevalence of myopia ≤−0.75 D was 16.5% (95% CI 13.9–19.4). The age standardised prevalence of myopia ≤−0.5 D was 39.6%, 95% CI (38.6–40.6) and the pattern with age was very similar to that observed for myopia ≤−0.75 D.

**Figure 2:**
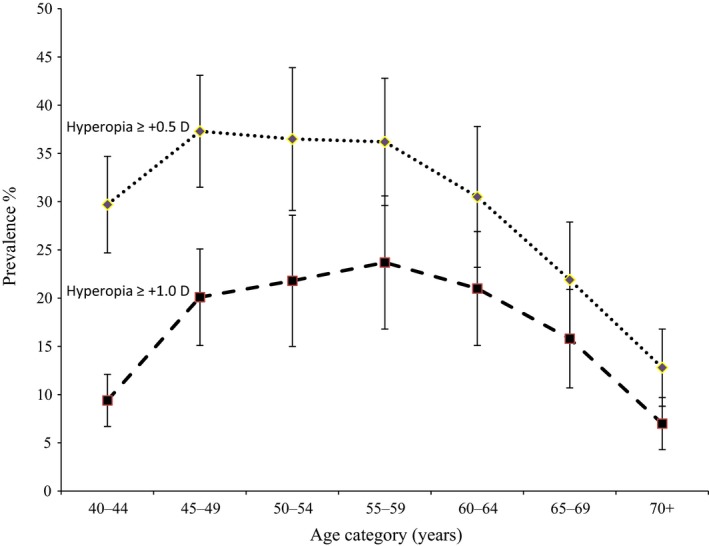
Prevalence of hyperopia with 95% CI by age for different cut points of spherical equivalent.

The age‐standardised prevalence of hyperopia was 30.3%, (95% CI: 29.2–31.3) and 17.0% (95% CI 16.3–17.8) for SE ≥+0.5 D and SE ≥+1.0 D respectively. Both categories of hyperopia were more common in women than men. Age‐standardised rates of hyperopia ≥+0.5 D and ≥+1.0 D in women were 32.6%, 95% CI, 31.1–34.0 and 19.7%, 18.6–20.5. Respective figures in men were 27.3% 95% CI, 25.9–28.7 and 13.9%, 13.0–14.9. The prevalence of both categories of hyperopia showed an initial increase from ages 40 to 44 years to ages 45 to 49 years with little or no further increase with age and a decline from the ages of 55 to 59 years (*Figure* 2).

**Figure 3:**
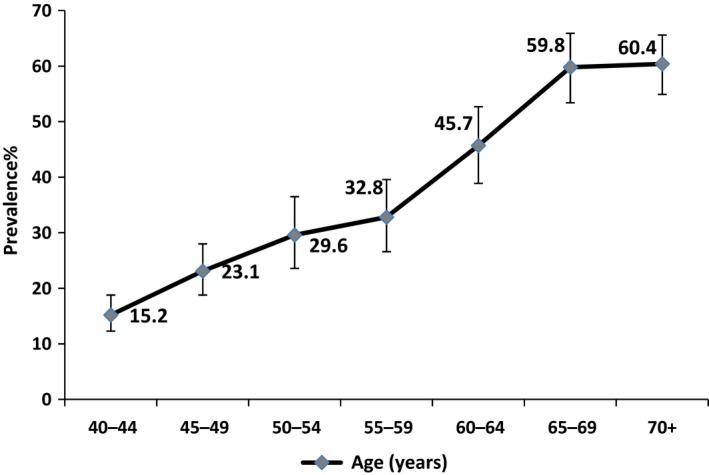
Prevalence of astigmatism (<−0.5 cylinder) by age with 95% CI.

The age‐standardised prevalence of astigmatism was 32.6% (29.3–36.1) and similar in men (32.1%, 95% CI, 28.0–35.5) and women, (33.1%, 29.3–37.0). Astigmatism prevalence rose with age up to the 65–69 age group with no further increase in those aged 70 years and over (*Figure* 3).

Presenting and best corrected VA in the better eye of people with myopia according to advanced cataract status are shown in *Table* 1. Seventy nine per cent of those (*n* = 1046) with myopia and advanced cataract had presenting vision worse than 6/18; of these, after best correction, 44% improved to 6/12 or better, 30% to less than 6/12 and equal or better than 6/18 and 27% remained with VA less than 6/18. In those with myopia without advanced cataract (*n* = 440), there was a lower proportion with presenting VA less than 6/18 (57%). After best correction, only 9% of these remained with VA less than 6/18, 22% improved to 6/12 to 6/18, and 69% had vision 6/12 or better. We also carried out a further analysis in myopes using individual eyes and tabulated presenting and corrected VA in the same eye categorised by advanced cataract in the corresponding eye (*Table* S1). Of 1860 eyes with advanced cataract, 86% had presenting VA less than 6/18 (*n* = 1595); of these, 35% (*n* = 558) remained with VA less than 6/18 after best correction. In 1063 eyes without advanced cataract, equivalent figures were 65% (*n* = 692) with presenting VA less than 6/18, of which 13% of eyes (*n* = 93) remained with VA less than 6/18 after best correction.

**Table 1:** Distribution of presenting and best corrected visual acuity in the better eye for people with myopia (SE ≤ −0.75 D) categorised by the presence or absence of advanced cataract^a^

	Best corrected VA							Best corrected VA					
	People with advanced cataract							People without advanced cataract					
Presenting VA	≥6/6, *n* (%)	<6/6–≥6/12, *n* (%)	6/12–≥6/18, *n* (%)	<6/18–≥6/60, *n* (%)	<6/60–≥3/60, *n* (%)	<3/60, *n* (%)	Total, *n* (%)	≥6/6, *n* (%)	<6/6–≥6/12, *n* (%)	6/12–≥6/18, *n* (%)	<6/18–≥6/60, *n* (%)	<6/60–≥/60, *n* (%)	Total, *n* (%)
≥6/6	6 (15.4)	1 (0.2)	0	0	0	0	7 (0.7)	11 (16.9)	0	0	0	0	11 (2.5)
<6/6–≥6/12	21 (53.8)	72 (13.7)	0	0	0	0	93 (8.9)	38 (58.5)	65 (22.0)	0	0	0	103 (23.4)
<6/12–≥6/18	9 (23.1)	96 (18.2)	15 (5.7)	0	0	0	120 (11.5)	9 (13.8)	65 (22.0)	1 (1.8)	1 (4.3)	0	76 (17.3)
<6/18–≥6/60	3 (7.7)	285 (54.1)	143 (54.8)	60 (28.2)	0	0	491 (46.9)	7 (10.8)	140 (47.3)	35 (63.6)	5 (21.7)	0	187 (42.5)
<6/60–≥3/60	0	67 (12.7)	77 (29.5)	108 (50.7)	1 (20.0)	0	253 (24.2)	0	25 (8.4)	14 (25.5)	14 (60.9)	0	53 (12.0)
<3/60	0	6 (1.1)	26 (10.0)	45 (21.1)	4 (80.0)	1 (100)	82 (7.8)	0	1 (0.3)	5 (9.1)	3 (13.0)	1 (100)	10 (2.3)
Total	39 (100)	527 (100)	261 (100)	213 (100)	5 (100)	1 (100)	1046 (100)	65 (100)	296 (100)	55 (100)	23 (100)	1 (100)	440 (100)

a Advanced cataract defined as LOCS III grade of nuclear ≥4, cortical ≥3, posterior subcapsular (PSC) ≥2, or dense opacities in either eye.

The distribution of presenting VA among people with hyperopia was 6/12 or more (59%); less than 6/12 and equal or better than 6/18 (18%); less than 6/18 and greater than or equal to 6/60 (21%); and less than 6/60 (1%) (data not shown). After correction, 56.7% had best corrected VA 6/6 or better and 42.4% had VA less than 6/6 and better or equal to 6/12.

In multivariable logistic regression analysis excluding participants with advanced cataract, all categories of myopia ≤−0.75 D were associated with increasing age (*Table* 2). Compared to those with no education, people with education up to secondary level were less likely to have low or moderate/high myopia (all odds ratios (OR) around 0.50). People with college education also had similar results for low myopia but a high OR (1.83) for moderate/high myopia; however the 95% CI were very wide, (0.37–9.03), reflecting the small numbers in this category. Those who spent longer hours outdoors daily were more likely to have either low or moderate/high myopia. There was a twofold increased OR for nuclear opacity score and low myopia (OR: 2.54; 95% CI: 1.90–3.41) and even higher for moderate/high myopia (OR: 4.19 95% CI: 2.30–7.64). Current but not past tobacco use was strongly associated with myopia compared to those who never used tobacco; the OR for moderate/high myopia (2.98; 95% CI: 1.90–4.68) was almost double that of low myopia (1.58; 95% CI: 1.05–2.36). Increasing height was associated with myopia but there was no association by gender, rural or urban place of residence, socio‐economic status or diabetes.

**Table 2:** Factors associated with myopia and grade of myopia in participants without advanced cataract^a^

Factors	All Myopia ≤−0.75 D 432 myopia vs 863 no RE			Low myopia ≤−0.75 to >−3 D 328 myopia vs 863 no RE			Moderate/high myopia ≤−3 D 104 myopia vs 863 no RE		
Distribution (%) or (mean) in all myopia	Odds ratio	95% CI	*p*	Odds ratio	95% CI	*p*	Odds ratio	95% CI	*p*
Age (54)	1.08	1.06–1.10	10^−14^	1.08	1.06–1.10	10^−14^	1.09	1.05–1.12	10^−5^
Women vs Men (43%)	1.35	0.88–2.09	0.17	1.22	0.75–1.97	0.42	1.68	0.72–3.90	0.23
Rural vs Urban (64%)	1.18	0.78–1.78	0.43	1.17	0.76–1.81	0.48	1.35	0.70–2.59	0.37
Socio‐economic score									
Lowest									
I (29%)	1			1			1		
II (21%)	1.07	0.70–1.65	0.74	1.16	0.69–1.94	0.58	0.97	0.67–1.41	0.89
III (27%)	1.23	0.81–1.89	0.33	1.31	0.79–2.17	0.30	0.96	0.58–1.61	0.89
Highest									
IV (23%)	1.04	0.66–1.64	0.86	1.16	0.68–1.99	0.59	0.47	0.25–0.89	0.02
*p* trend			0.71			0.50			0.04
Education									
None (38%)	1			1			1		
Elementary (34%)	0.62	0.44–0.88	0.01	0.61	0.42–0.88	0.01	0.50	0.25–0.98	0.05
Secondary (22%)	0.57	0.32–1.02	0.06	0.49	0.27–0.89	0.02	0.70	0.23–2.10	0.52
College (6%)	0.70	0.34–1.42	0.32	0.50	0.29–0.88	0.02	1.83	0.37–9.03	0.46
*p* trend			0.04			0.003			0.68
Daily hours outside (2.9)	1.17	1.08–1.26	<0.001	1.16	1.07–1.26	<0.001	1.23	1.08–1.39	0.002
Nuclear opacity^b^ (2.8)	2.65	2.00–3.51	10^−11^	2.54	1.90–3.41	10^−9^	4.19	2.30–7.64	10^−5^
Height (cm) (157)	1.02	1.00–1.05	0.04	1.03	0.99–1.05	0.06	1.03	0.99–1.06	0.11
Diabetes^c^ (6%)	1.09	0.56–2.11	0.79	1.21	0.61–2.41	0.59	0.84	0.26–2.70	0.77
Tobacco use									
Never (58%)	1			1			1		
Past (7%)	1.00	0.56–1.80	0.99	1.09	0.57–2.08	0.80	0.61	0.18–2.03	0.42
Current (35%)	1.76	1.27–2.45	0.001	1.58	1.05–2.36	0.03	2.98	1.90–4.68	10^−5^

a Advanced cataract defined as LOCS III grade of nuclear ≥4, cortical ≥3, posterior subcapsular (PSC) ≥2, or dense opacities.

b LOCS III grading of nuclear opacity ≤4.

c Random blood glucose of 200 mg dL^−1^ or above.

We found significant interactions of age group with level of education and with time spent outdoors (*Table* 3). Whereas there was an inverse trend of lower ORs for myopia with increasing levels of education in the older age group, the opposite relationship with education was found for the younger age group. In the younger age group, those with college level education had a nearly twofold increased odds of myopia compared to an odds of 0.28 in the older age group (*p* interaction = 0.001). The distribution of education was very different between the two age groups: 29% of the younger age group had secondary education or above compared to 13% of those aged above 60 years. Increasing hours per day spent outdoors was associated with higher odds of myopia, OR = 1.19, in those aged 60 years and over and no increased OR (1.01) in the younger age group (*p* interaction = 0.04). Mean daily time outdoors was 2.5 (S.D. = 1.7) hours in the younger age group compared to 3.5 (S.D. = 2.4) hours in the older age group.

**Table 3:** Association of education and time spent outdoors with myopia by age group (less than 60 years or 60 years and over)

	Age <60 93 myopia vs 601 with no RE			Age 60+ 339 myopia vs 262 with no RE			
All Myopia ≤−0.75 D	Odds ratio^a^	95% CI	*p*	Odds ratio^a^	95% CI	*p*	*p* Interaction
Education							
None	1			1			
Elementary	1.10	0.70–1.73	0.67	0.45	0.29–0.71	0.001	0.003
Secondary	1.25	0.63–2.48	0.52	0.36	0.17–0.76	0.007	0.01
College	1.94	0.81–4.63	0.14	0.28	0.13–0.58	0.001	0.001
*p* trend			0.15			0.001	

	Odds ratio^b^	95% CI	*p*	Odds ratio^b^	95% CI	*p*	*p* Interaction
Daily hours outdoors	1.01	0.88–1.15	0.93	1.19	1.08–1.32	0.001	0.04

a Adjusted for age, gender, urban/rural, socio‐economic status, average daily hours outdoors, height, nuclear opacity score, diabetes, tobacco use.

b Adjusted for age, gender, urban/rural, socio‐economic status, education, nuclear opacity score, height, diabetes, tobacco us.

Results of multivariable logistic regression for hyperopia were similar irrespective of the presence or absence of advanced cataract (*Table* 4). Increasing age and female gender were associated with hyperopia. There was a clear trend for increasing ORs with increasing levels of education with college education being associated with a nearly twofold OR. Nuclear opacity score, increasing daily time spent outdoors, rural place of residence, and current tobacco use were significantly associated with odds ratios of less than one. There was no association with socio‐economic status, height or diabetes with hyperopia.

**Table 4:** Factors associated with hyperopia in participants with and without advanced cataract^a^

Factors	Hyperopia ≥+1 D					
	With advanced cataract 551 hyperopia vs 1169 with no RE			Without advanced cataract 432 hyperopia vs 863 with no RE		
Distribution (%) or (mean) in all hyperopia	Odds ratio	95% CI	*p*	Odds ratio	95% CI	*p*
Age (56)	1.06	1.05–1.08	10^−15^	1.08	1.06–1.10	10^−19^
Women vs Men (54%)	1.86	1.23–2.83	0.003	1.62	1.04–2.51	0.03
Rural vs Urban (51%)	0.69	0.51–0.94	0.02	0.71	0.52–0.98	0.04
Socio‐economic score						
Lowest						
I (23%)	1			1		
II (21%)	1.35	0.91–1.99	0.14	1.18	0.83–1.69	0.36
III (29%)	1.18	0.77–1.80	0.45	1.03	0.66–1.61	0.89
Highest						
IV (28%)	1.35	0.90–2.03	0.15	1.26	0.86–1.85	0.24
*p* trend						0.35
Education						
None (32%)	1	1		1		
Elementary (36%)	1.30	1.01–1.66	0.04	1.15	0.86–1.54	0.34
Secondary (24%)	1.61	1.13–2.30	0.01	1.44	0.91–2.28	0.12
College (8%)	1.85	1.16–2.96	0.01	1.78	1.06–3.00	0.03
*p* trend						0.04
Daily hours outside (2.3)	0.89	0.81–0.96	0.01	0.85	0.78–0.94	0.001
Nuclear opacity b (2.8)	0.56	0.50–0.62	10^−24^	0.66	0.57–0.77	10^−6^
Height (156)	1.00	0.98–1.01	0.61	0.99	0.97–1.01	0.45
Diabetes^c^(8%)	1.02	0.76–1.37	0.88	0.93	0.65–1.34	0.72
Tobacco use						
Never (69%)	1			1		
Past (7%)	0.98	0.66–1.45	0.92	1.03	0.60–1.76	0.92
Current (24%)	0.67	0.48–0.94	0.02	0.59	0.42–0.84	0.003

a LOCS III grade of nuclear ≥4, cortical ≥3, posterior subcapsular (PSC) ≥2, or dense opacities.

b LOCS III grading of nuclear opacity.

c Random blood glucose of 200 mg dL^−1^ or above.

A high proportion of people with astigmatism were myopic (64%). We therefore controlled for myopia in the logistic regression (*Table* 5). Age, rural residence, and nuclear opacity score were associated with increased odds of astigmatism and increasing education with lower odds. There was no association of cortical opacity score with astigmatism either when included in models with nuclear opacity (OR = 1.00, 95% CI 0.85–1.15; *Table* 5) or models without nuclear opacity (OR = 1.02, 0.87–1.20). Results for myopia were little changed by controlling for astigmatism; for example, daily hours outside was associated with an OR of 1.17, 1.08–1.26 (*Table* 3) and an OR of 1.19, 1.10–1.28 in analyses controlled for astigmatism (data not shown).

**Table 5:** Factors associated with astigmatism (<−0.5 cylinder) in participants without advanced cataract^a^

Factors	Astigmatism (<−0.5 cylinder) 531 astigmatism vs 1193 no astigmatism		
Distribution (%) or (mean)	Odds ratio	95% CI	*p*
Age (55)	1.05	1.03–1.06	10^−5^
Women vs Men (48%)	0.73	0.45–1.18	0.20
Rural vs Urban (57%)	1.53	1.10–2.12	0.01
Socio‐economic score			
Lowest			
I (25%)	1		
II ((21%)	1.08	0.73–1.60	0.70
III (28%)	0.67	0.44–1.01	0.06
Highest			
IV (26%)	0.81	0.59–1.11	0.20
*p* trend			0.07
Education			
None (33%)	1	1	
Elementary (35%)	0.88	0.62–1.26	0.49
Secondary (24%)	0.82	0.55–1.23	0.34
College (8%)	0.58	0.32–1.04	0.07
*p* trend			0.05
Daily hours outside (2.6)	1.03	0.96–1.11	0.39
Nuclear opacity^b^ (2.7)	1.41	1.16–1.71	<0.0001
Cortical opacity^b^(0.72)	1.00	0.85–1.18	0.96
Height (cm) (157)	0.99	0.97–1.02	0.57
Diabetes^c^ (7%)	1.08	0.63–1.84	0.79
Tobacco use			
Never (64%)	1		
Past (6%)	1.03	0.62–1.73	0.90
Current (30%)	0.85	0.57–1.27	0.42
Myopia (≤−0.75 D) (25%)	11.2	7.87–16.0	10^−12^

a LOCS III grade of nuclear ≥4, cortical ≥3, posterior subcapsular (PSC) ≥2, or dense opacities.

b Mean LOCS III nuclear opacity score.

Mean LOCS III cortical opacity score.

c Random blood glucose of 200 mg dL^−1^ or above.

## Discussion

The prevalence of myopia in our study was similar to two recent populationbased studies in India using similar methods, the Chennai Glaucoma Study^21^ and the Andhra Pradesh Eye Disease Study (APEDS),^9^ respectively. Defining myopia as SE < −0.5 D, both studies show increasing prevalence of myopia with age and similar prevalence rates to our study with some minor variation in the oldest age groups (age 70+ years). In contrast, in the Singapore Indian Eye Study (SINDI)^31^ of people aged 40–80 years, the prevalence of myopia decreased with age with a minor increase in the oldest age group. In the youngest age group (40–49 years) the prevalence rate was higher (33.3%; 95% CI: 30.2–36.4) compared to 22.3% (95% CI: 18.5–26.5) in the INDEYE study and 19.2% and 15.7% in the APEDS and the Chennai Glaucoma study, respectively.

In another similar racial and cultural population in Bangladesh,^7^ prevalence rates of myopia (SE ≤ −0.5 D) were lower in the younger age groups (age 40–59 years) but comparable to the Indian studies in the oldest age groups with a steady increase in prevalence from the age group of 40–49 years onwards. In the Singapore Chinese study,^12^ the rates of myopia fell with increasing age with higher rates in the youngest age group (40–49 years) compared to the Indian studies. In the Singapore Malay Eye Survey,^32^ the rates also decreased with age but increased in the 70–80 year age group; the rates in men for 70–80 year olds were 36.9% (95% CI: 31.4–42.6) compared with 16.0% (95% CI: 12.2–20.4) in the 60–69 years age group. A similar pattern was observed in the Tanjong Pagar study of Singapore Chinese.^12^

In a pooled analysis of results from population based studies in the United States, Australia and Europe,^5^ prevalence rates of myopia (SE ≤ −1.0 D) consistently followed a pattern of high rates at younger ages compared to middle aged and older age groups, with a small increase in the 80+ years age group. The rates varied by ancestry. Compared to people of European ancestry, prevalence rates were lower in African Americans especially at young ages. Hispanics had rates intermediate between African Americans and Europeans. Prevalence rates would have been higher had a definition (SE ≤ −0.5 D) been used. Compared to our study and others in India, prevalence rates were higher in those of European ancestry in the youngest age group (around 40%) using the definition of myopia (SE ≤ −1.0 D). A recent study based on pooling results on myopia (defined as SE ≤ −0.75 D) from 33 European studies confirmed the pattern of falling rates from age 40 years onwards, reaching a nadir at ages 65–90 years with a subsequent small increase in the oldest age group (90+ years).^10^

The most striking difference between studies is the pattern of decreasing myopia prevalence with increasing age observed in high income settings compared with increasing myopia with age in low income settings. The Los Angeles Latino Eye Study (LALES) showed a pattern of a small decrease in age specific prevalence of myopia (SE ≤ −0.5 D) from 40 to 49 years up to age 60 to 69 years, followed by an increase up to the age of 80+ years; this increase was most marked in men.^11^ The authors commented that the pattern observed in LALES was intermediate between the patterns observed in low and high income settings and might be explained by differences in the prevalence of nuclear opacities. Exclusion of nuclear cataract (LOCS II grade ≥2) in LALES considerably attenuated the rise in myopia prevalence after the age 60–69 years but did not reverse the relationship with age. In the Bangladesh study, also with very high rates of cataract, there was a pattern of falling myopia rates with age after exclusion of cataract.^7^ Similarly, in the SINDI study, the association between age and myopia was reversed from increasing rates with increasing age to falling rates with increasing age after exclusion of cataract.^31^ In our study, however, exclusion of advanced cataract reduced the prevalence of myopia by a half but did not alter the pattern of increasing prevalence with increasing age. This difference might be explained by the older age of our population and the presence of mild nuclear opacities even after exclusion of advanced cataract. Nuclear opacification leading to myopic shift has been postulated as an explanation for the rise in myopia with increasing age and the strong association with myopia and nuclear opacities observed in many studies^7^, ^11^, ^33^, ^34^, ^35^ including our study and other studies in India.^9^, ^21^ The findings from these studies and our own emphasise the adverse effects of cataract on visual acuity and refractive errors in many low income populations and highlight the need to address low access to eye care in many populations.^36^

There is good evidence that less time spent outdoors is a risk factor for myopia in children and young adults.^18^, ^19^ Conversely, the long term adverse effect of time spent outdoors in populations exposed to high levels of UV radiation is to increase the risk of cataract.^37^ In our study we found that increasing hours per day spent outdoors was associated with higher odds of myopia in those aged 60 years and over and no association in the younger age group. Since these associations were controlled for nuclear and cortical opacities, it is not clear why time spent outdoors would be associated with myopia in older people other than through nuclear shift. However, we cannot exclude other unmeasured factors that could be confounders.

Similar to the other population‐based studies in adults described above, we used non‐cycloplegic refraction which has been considered to be a valid method of measuring refraction in adults aged 40 years and over.^12^, ^31^ Results from the population‐based Tehran study, which systematically compared cycloplegic and non‐cycloplegic measurements, reported that the greatest differences in the methods were observed for hyperopia below the age of 40 years (mean difference 0.7–0.8 D) with smaller differences in the 40–50 years age group (mean difference 0.45 D).^38^ The differences in the two methods for myopia were very small especially in the 50 years and over age group. While our estimates for hyperopia may suffer from some degree of measurement error especially in the youngest age group, we do not consider this is sufficient to materially bias our results for hyperopia.

We observed poorer visual acuity and less improvement after best correction in myopes with advanced cataract compared to myopes without cataract. These findings were similar whether we used an analysis by person or by eye. The clinical recommendation for an individual patient with advanced cataract and myopia requires consideration of many factors: the degree of myopia and astigmatism, the severity of advanced cataract, the best distance visual acuity attainable by refractive correction, functional problems and vision‐related quality of life as reported by the patient. In addition, near vision and near vision activities such as reading and sewing may be prioritised by some patients with low myopia without significant astigmatism over distance acuity.

In common with one other study in India and other studies in similar low income settings, we found that increasing levels of education were associated with lower rates of myopia.^7^, ^8^, ^9^ Our risk factor analysis excluded participants with advanced cataract and further adjusted for a score of nuclear opacity. In contrast, after the exclusion of advanced cataract in the Bangladesh study,^7^ there was no association between myopia and levels of education other than for college education, which was associated with a nearly twofold increased ORs for all levels of myopia. We found highly significant interactions between age and education. In the age groups above 60 years, increasing levels of education were associated with decreasing ORs while in the age groups less than 60 years, there was a trend for increasing ORs with education. College education was associated with a nearly twofold OR of myopia although the statistical evidence was weak (*p* = 0.14). Secular trends in education were reflected in our study with twice as many younger people in secondary and college education compared to the older age groups. The Handan Eye Study^13^ found high school education and above was associated with a twofold OR of myopia in those aged 50 years and above but there was no relationship with education in those aged under 50 years. The Singapore Eye Study reported that increasing education level was a risk factor for myopia in Singapore Chinese^12^ and Malays,^39^ but not in the Singapore Indian population.^31^ However, increased time spent on reading and writing was a risk factor for myopia in Singapore Indians. The difference in risk factors between Indians in India and Indians in Singapore (predominantly migrants from the southern states of India) probably reflects higher levels of education and other factors related to migration.^31^ Second generation Singapore Indians had a higher prevalence of myopia compared to first generation immigrants, possibly reflecting higher levels of education and increased axial length^4^

In a recent study of urban school children in Delhi, increasing time spent on near activities (reading, computer games) were risk factors for myopia and greater time spent outdoors was protective.^40^

Other risk factors for myopia identified in our study were height and current tobacco use. The association with height as a measure of axial length was in agreement with other studies^22^, ^31^ and remained after adjustment for socio‐economic status. However, we accept the limitations on using height as a proxy for axial length. Current use of tobacco, but not past use, was associated with a nearly twofold OR of myopia even after adjustment for nuclear opacities. Past but not current smoking was reported to be associated with myopia in APEDS^9^ and as protective in people under 50 years in the Handan Eye Study.^13^

We found an initial increase of hyperopia prevalence with little change until the age of 50 years, after which it decreased. The pattern of initial increase followed by decrease (after 70 years) in hyperopia prevalence with age has been reported in studies published from India,^9^, ^21^ Bangladesh^7^ and Singapore,^12^, ^31^ reflecting the onset of lenticular myopia due to nuclear sclerosis. We found the association of risk factors for hyperopia was in the opposite direction of those for myopia. Prevalence of hyperopia was higher among women compared to men, in agreement with studies in a number of settings^2^, ^5^, ^7^, ^12^ including India.^9^, ^21^ In our study, increasing education and less time spent outdoors were associated with hyperopia. These relationships did not differ by cataract status.

### Study limitations

Our response rate was 72%. The participants were older than non‐participants but there were no differences between participants and non‐participants in gender and education. However, we did not have information on other factors in non‐participants to assess any possible biases.

Our study was cross‐sectional and therefore we were unable to establish causal relationship between the associations we observed in the risk factor analyses. While education and outdoor exposure at young age are exposures occurring before the measurement of refractive errors measured in adulthood, they may be affected by recall bias. Other variables such as height, diabetes and socio‐economic status are contemporary with refractive error measures and may not reflect earlier exposures. Apart from education, we had no information on childhood exposures of the participants including outdoor exposure during childhood. Although our models controlled for key confounders, uncontrolled confounding may still be present due to unmeasured variables.

There may be errors in our measures of refractive errors and visual acuity. Similar to other population‐based studies in adults described above, we used non‐cycloplegic refraction which has been considered to be a valid method of measuring refraction in adults aged 40 years and over.^12^, ^31^ Results from the population‐based Tehran study which systematically compared cycloplegic and non‐cycloplegic measurements reported that the greatest differences in the methods were observed for hyperopia below the age of 40 years (mean difference 0.7–0.8 D) with smaller differences in the 40–50 years age group (mean difference 0.45 D).^38^ The differences in the two methods for myopia were very small especially in the 50 years and over age group. While our estimates for hyperopia may suffer from some degree of measurement error especially in the youngest age group, we do not consider this is sufficient to materially bias our results for hyperopia. VA testing and refraction was performed by two optometrists who had been additionally trained for this study and followed a structured manual of operations. However, we did not perform any formal assessment of intra‐ or inter‐observer variations. In contrast, the lens opacity measurement and grading were subject to extensive quality assurance as described in detail elsewhere.^23^

In conclusion, our study provides further epidemiologic data on the prevalence of myopia, hyperopia and astigmatism in an adult population in southern India. Advanced cataract was associated with increased myopia prevalence in our study population.

## Disclosure

The authors report no conflicts of interest and have no proprietary interest in any of the materials mentioned in this article.

## Supporting information


**Table S1.** Distribution of presenting and best corrected visual acuity in eyes of people with myopia (SE ≤ −0.75 D) categorized by the presence or absence of advanced cataract in the corresponding eye.
