# Supplementary material for: Prevalence and risk factors for myopia and other refractive errors in an adult population in southern India
Source: Ophthalmic Physiol Opt. 2018 Mar 25;38(3):346–58. doi: 10.1111/opo.12447 (PMC6001660; doi:10.1111/opo.12447)
Supplement: Supplementary file 1 — Supplementary file (PDF 564 KB) [file 44402_2018_3803016_MOESM1_ESM.pdf]

Supplemental Table. Distribution of presenting and best corrected visual acuity in eyes of people with myopia (SE≤ -0.75 D) categorized by the presence or absence of advanced cataract in the corresponding eye[1]

	Best Corrected VA							Best Corrected VA				
	Eyes with advanced cataract						Eyes without advanced cataract					
Presenting VA	>=6/12	<6/12->=6/18	<6/18->=6/60	<6/60->=3/60	<3/60	Total	>=6/12	<6/12->=6/18	<6/18->=6/60	<6/60->=3/60	<3/60	Total
>=6/12	108	0	0	0	0	108<br>5.8%	203	0	0	0	0	203<br>19.1%
<6/12->=6/18	128	29	0	0	0	157<br>8.4%	163	4	1	0	0	168<br>15.8%
<6/18->=6/60	453	269	145	0	0	867<br>46.6%	374	97	18	0	0	489<br>46.0%
<6/60->=3/60	109	141	246	5	0	501<br>26.9%	60	47	54	0	0	161<br>15.1%
<3/60	13	52	110	12	40	227<br>12.2%	6	15	15	3	3	42<br>4.0%
Total Eyes	811	491	501	17	40	1860	806	163	88	3	3	1063

[1] advanced cataract defined as LOCS III grade of nuclear ≥4, cortical ≥3, posterior sub-capsular (PSC) ≥2, or dense opacities
